# Prevalence and risk factors for scrotal lesions/anomalies in a cohort of Brazilian men ≥ 40 years of age

**DOI:** 10.1590/1516-3180.2014.1322495

**Published:** 2014-04-01

**Authors:** Frederico Ramalho Romero, Antonio Wilson Romero, Rui Manuel de Sousa Sequeira Antunes de Almeida, Fernando Cesar de Oliveira, Renato Tambara

**Affiliations:** I MD, MSc, PhD, Department of Surgery, Discipline of Urology, Universidade Federal do Paraná (UFPR), Curitiba, Paraná, Brazil; II MD. Urologist, Urology Service, Hospital Policlínica, Cascavel, Paraná, Brazil; III MD, MSc, PhD. Coordinator, College of Medicine, Faculdade Assis Gurgacz (FAG), Cascavel, Paraná, Brazil; IV MD, MSc, Coordinator, Núcleo de Atenção à Saúde, Instituto Curitiba de Saúde (ICS), Curitiba, Paraná, Brazil; V MD, MSc, PhD. Professor, Department of Surgery, Discipline of Urology, Universidade Federal do Paraná (UFPR), Curitiba, Paraná, Brazil

**Keywords:** Epidemiology, Etiology [subheading], Prevalence, Risk factors, Genital diseases, male, Epidemiologia, Etiologia, Prevalência, Fatores de risco, Doenças dos genitais masculinos

## Abstract

**OBJECTIVE:**

To estimate the prevalence of and risk factors for cutaneous lesions of the scrotum and intrascrotal lesions/anomalies among men included in a prostatic cancer screening program in a Brazilian metropolitan city.

**DESIGN AND SETTING:**

Cross-sectional study, private outpatient healthcare service.

**METHODS:**

1731 men aged 40 years or over, participating in a prostate cancer screening program conducted by the municipal public employees' healthcare system, underwent systematic urological assessment by a single examiner.

**RESULTS:**

The prevalence of scrotal diseases in our sample was 44.7% (773/1731). *Tinea cruris* occurred in 203 (11.7%) of the participants, with higher risk among diabetics and lower prevalence among nonwhite individuals; scrotal tinea in eight (0.5%), with higher risk among hypertensive men; subcutaneous nodules in 12 (0.7%), especially in individuals with low schooling level; hydrocele in 283 (16.4%), with higher frequency among participants over the age of 60 years, diabetics or individuals with previous histories of nonspecific urethritis; spermatoceles in 174 (10.1%), with greater prevalence among individuals over the age of 60 years or diabetics, and lower frequency among individuals who underwent vasectomy; unilateral testicular hypotrophy/atrophy in 167 (9.7%) and bilateral hypotrophy/atrophy in 93 (5.4%), both occurring more frequently in individuals over the age of 60 years; absence of palpable testicles due to cryptorchidism in 7 (0.4%); and epididymitis/orchitis in 5 (0.3%), with higher prevalence among diabetics. No cases of cancer were identified in this sample.

**CONCLUSIONS:**

Scrotal diseases were highly prevalent in this population of Brazilian men.

## Abstract (pt)

**OBJETIVO:**

Estimar a prevalência e fatores de risco de lesões cutâneas do escroto e de lesões/anomalias intraescrotais entre participantes de programa de rastreamento para câncer de próstata em uma cidade metropolitana brasileira.

**TIPO DE ESTUDO E LOCAL:**

Estudo transversal, serviço privado de atendimento ambulatorial à saúde.

**MÉTODOS:**

1.731 homens com idade igual ou superior a 40 anos, participantes do programa de rastreamento de câncer de próstata conduzido pelo sistema de saúde dos funcionários públicos municipais, foram submetidos à avaliação urológica sistemática por um único examinador.

**RESULTADOS:**

A prevalência de doenças escrotais nossa amostra foi de 44.7% (773/1731). *Tinea cruris* ocorreu em 203 (11,7%) dos participantes, com maior risco em diabéticos e menor prevalência em indivíduos não brancos; tinea escrotal em oito (0,5%), com maior risco em homens hipertensos; nódulos subcutâneos em 12 (0,7%), especialmente em indivíduos com baixa escolaridade; hidrocele em 283 (16,4%), com maior frequência nos participantes com mais de 60 anos, diabetes ou história prévia de uretrite inespecífica; espermatoceles em 174 (10,1%), com maior prevalência acima dos 60 anos de idade ou com diabetes, e menor frequência naqueles submetidos a vasectomia; hipotrofia/atrofia testicular unilateral em 167 (9,7%) e hipotrofia/atrofia bilateral em 93 (5,4%), ambas ocorrendo mais frequentemente nos indivíduos com mais de 60 anos; ausência de testículos palpáveis devido à criptorquidia em 7 (0,4%); e epididimite/orquite em 5 (0,3%), com prevalência aumentada em diabéticos. Não foram identificados casos de câncer nesta amostra.

**CONCLUSÕES:**

As doenças escrotais foram altamente prevalentes nesta população de homens brasileiros.

## INTRODUCTION

Cutaneous lesions of the scrotum and intrascrotal lesions/anomalies are common findings in the adult male population. The prevalence of these lesions is difficult to determine accurately, given that they are frequently oligosymptomatic and are often discovered incidentally during physical examination. The results differ according to the patients' ages, racial influences, geographic locations, comorbidities and socioeconomic status.^1^

The setting in which the study is conducted (based in the population/community or hospital/clinic setting), the type of study (retrospective or prospective) and the type of diagnostic assessment (clinical, laboratory or imaging-based) may also influence prevalence levels.^2^

Epidemiological studies are important because they contribute towards taking an appropriate approach to the conditions, improving awareness, promoting educational practices and preventive measures and expediting treatment. They may also enable comparisons within and between countries, allow for temporal variations between different ages and time periods, and guide future research to evaluate the pathogenesis, etiology and risk factors of these diseases.

## OBJECTIVE

The objective of this paper was to report on the overall prevalence and risk factors of cutaneous lesions of the scrotum and intrascrotal lesions/anomalies collected prospectively in a crosssectional study conducted in a Brazilian metropolitan city.

## METHODS

Between December 2006 and April 2011, 1731 subjects were included in this investigation. The participants were men aged 40 years or over who were undergoing outpatient evaluation as part of a free prostate cancer screening program conducted by the municipal public employees' healthcare system (Curitiba Institute of Health), which is a private institution directed exclusively towards workers employed by the municipality of Curitiba. The study protocol was reviewed and approved by the Institutional Ethics Committee for Human Research (registry number 2253.147/2010-06).

During evaluation, the participants were classified by a single examiner as white or nonwhite (including brown or black); they answered a general questionnaire that asked about their age, schooling level and personal histories of diabetes, arterial hypertension, nonspecific urethritis or vasectomy (Table 1); and were offered a complete genital-pelvic examination. 

**Table 1.** Demographic and clinical characteristics of study population

Characteristic	Number	Percentage (%)
Age		
< 60 years	1352	78.1
≥ 60 years	379	21.9
Total	1731	100.0
Schooling level		
Elementary school or lower	647	37.4
High school or higher	937	54.1
Missing data	147	8.5
Skin color		
White	643	37.1
Nonwhite (black or brown)	588	34.0
Missing data	500	28.9
Arterial hypertension		
Yes	595	34.4
No	1133	65.4
Missing data	3	0.2
Diabetes *mellitus*		
Yes	180	10.4
No	1548	89.4
Missing data	3	0.2
Past history of nonspecific urethritis		
Yes	497	28.7
No	1012	58.5
Missing data	222	12.8
Past history of vasectomy		
Yes	210	12.1
No	1518	87.7
Missing data	3	0.2

The urological examination was performed in a standardized manner, on all subjects in the supine position by the same examiner. The scrotum was initially inspected for cutaneous lesions, and was then palpated to detect any intrascrotal lesions/anomalies, including hydrocele, spermatocele, testicular hypotrophy/atrophy, testicular cancer or absence of a palpable testis. Results were collected during the first evaluation on each patient, and were not computed more than once for each participant.

The outcomes of interest included the prevalence of scrotal lesions/anomalies, and the relative risk (RR) and 95% confidence intervals (95% CI) of the lesions/anomalies, according to age (≥ 60 versus < 60 years), schooling level (elementary school or lower versus high school or higher), skin color (nonwhite versus white), personal history of diabetes, arterial hypertension, nonspecific urethritis and vasectomy (yes versus no, for all of these last four categories). 

Statistics for categorical variables were calculated using Fisher's exact test or Pearson's chi-square test, as appropriate, and statistical significance was defined as situations in which P < 0.05 or when the 95% CI did not include the null hypothesis (95% CI ≠ 1.00).

## RESULTS

Cutaneous lesions of the scrotum were identified in 230 participants (13.3%, 230/1731). Mycosis was present in 211 individuals (91.7%, 211/230), and 203 of these cases were Tinea cruris (88.3% of all cutaneous lesions on the scrotum [203/230] and 11.7% of all participants [203/1731]). Fifty-nine participants (29.0%, 59/203) with Tinea cruris presented associated balanoposthitis. Tinea cruris occurred more commonly in participants with diabetes (RR = 1.55, P < 0.05), and less frequently in those of nonwhite skin color (RR = 0.68, P < 0.05) (Table 2). Scrotal tinea was identified in eight men (3.5% [8/230] or 0.5% [8/1731]), and two of these (25%, 2/8) presented associated balanoposthitis or Tinea cruris. Scrotal tinea was more prevalent in men with arterial hypertension than in those without arterial hypertension (RR = 5.71, P < 0.05) (Table 2).

**Table 2.** Prevalence of scrotal diseases and relative risks (RR) and 95% confidence intervals (CI) of the lesions/anomalies, according to age, schooling level, skin color and histories of diabetes mellitus, arterial hypertension, nonspecific urethritis and vasectom

	Age: ≥ 60 vs. < 60; RR; 95% CI	Schooling level: elementary school vs. high school or higher; RR; 95% CI	Skin color: nonwhite vs. white; RR; 95% CI	Diabetes: yes vs. no; RR; 95% CI	Arterial hypertension: yes vs. no; RR; 95% CI	History of nonspecific urethritis; yes vs. no; RR; 95% CI	Vasectomy: yes vs. no; RR; 95% CI
*Tinea cruris*	13.2 vs. 11.3 1.16 0.86-1.57	11.7 vs. 11.6 1.01 0.77-1.33	9.5 vs. 14.0 (*) 0.68 0.50-0.93	17.2 vs. 11.1 (*) 1.55 1.09-2.20	13.5 vs. 10.9 1.24 0.95-1.61	13.3 vs. 11.1 1.20 0.91-1.60	11.0 vs. 11.9 0.92 0.61-1.39
Scrotal tinea	0.5 vs. 0.4 1.19 0.24-5.87	0.3 vs. 0.4 0.72 0.13-3.94	0.2 vs. 0.3 0.55 0.05-6.01	1.1 vs. 0.4 2.87 0.58-14.10	1.0 vs. 0.2 (*) 5.71 1.16-28.22	0.2 vs. 0.5 0.41 0.05-3.48	0.0 vs. 0.5 0.00 0.00-NaN
Subcutaneous nodules	0.8 vs. 0.7 1.19 0.32-4.37	1.4 vs. 0.3 (*) 4.34 1.18-15.99	1.2 vs. 0.8 1.53 0.49-4.80	0.6 vs. 0.7 0.78 0.10-6.02	0.7 vs. 0.7 0.95 0.29-3.15	0.2 vs. 1.1 (*) 0.19 0.02-1.43	0.5 vs. 0.7 0.66 0.09-5.06
Hydrocele	24.5 vs. 14.1 (*) 1.74 1.40-2.17	17.7 vs. 15.7 1.13 0.91-1.41	15.3 vs. 16.2 0.95 0.73-1.23	33.0 vs. 16.5 (*) 2.01 1.62-2.49	18.3 vs. 15.3 1.19 0.96-1.48	19.9 vs. 14.8 (*) 1.35 1.07-1.69	12.9 vs. 16.8 0.77 0.53-1.11
Spermatocele	15.0 vs. 8.8 (*) 1.70 1.27-2.29	11.3 vs. 9.8 1.15 0.86-1.53	10.2 vs. 9.0 1.13 0.80-1.59	16.1 vs. 9.4 (*) 1.71 1.19-2.47	11.9 vs. 9.2 1.30 0.98-1.73	11.1 vs. 9.6 1.16 0.85-1.58	5.7 vs. 10.7 (*) 0.77 0.53-1.11
Unilateral testicular hypotrophy/atrophy	14.2 vs. 9.1 (*) 1.57 1.15-2.14	10.7 vs. 9.6 1.12 0.82-1.52	8.8 vs. 12.2 0.72 0.51-1.02	11.3 vs. 10.1 1.12 0.71-1.76	10.3 vs. 10.2 1.01 0.75-1.37	11.5 vs. 9.5 1.21 0.88-1.66	9.3 vs. 10.3 0.90 0.57-1.41
Bilateral testicular hypotrophy/atrophy	8.5 vs. 5.3 (*) 1.62 1.06-2.48	6.7 vs. 5.5 1.21 0.81-1.83	5.9 vs. 6.2 0.97 0.61-1.54	7.5 vs. 5.8 1.29 0.72-2.31	5.8 vs. 6.1 0.95 0.63-1.45	6.3 vs. 6.1 1.04 0.67-1.61	2.6 vs. 6.4 (*) 0.41 0.17-0.99
Epididymitis/orchitis	0.5 vs. 0.2 2.38 0.40-14.18	0.0 vs. 0.3 0.00 0.00-NaN	0.0 vs. 0.5 0.00 0.00-NaN	1.7 vs. 0.1 (*) 12.90 2.17-76.69	0.3 vs. 0.3 1.27 0.21-7.58	0.4 vs. 0.2 2.04 0.29-14.41	0.5 vs. 0.3 1.81 0.20-16.09
Cryptorchidism	0.5 vs. 0.4 1.43 0.28-7.33	0.6 vs. 0.2 2.90 0.53-15.77	0.7 vs. 0.3 2.19 0.40-11.90	0.0 vs. 0.5 0.00 0.00-NaN	0.5 vs. 0.4 1.43 0.32-6.36	0.4 vs. 0.5 0.81 0.16-4.18	0.5 vs. 0.4 1.20 0.15-9.96

* Statistically significant differences (Fisher's exact test or Pearson's chi-square test)

RR = relative risk

CI = confidence interval

NaN = not a number

vs . = versus

Other cutaneous lesions encountered on the scrotum included subcutaneous nodules (epidermal cysts) in 12 participants (5.2% [12/230] or 0.7% [12/1731]); psoriasis in one individual (0.4% [1/230] or 0.06% [1/1731]); and a nonspecific papillary rash in one individual (0.4% [1/230] or 0.06% [1/1731]). The risk-adjusted prevalence of these lesions demonstrated that subcutaneous nodules were more common in subjects with low schooling level (RR = 4.34, P < 0.05), and less prevalent in those with a history of nonspecific urethritis (RR = 0.19, P < 0.05) (Table 2).

Intrascrotal lesions/anomalies were identified in 638 participants (36.9%, 638/1731). Hydrocele was present in 283 participants (44.4% of all intrascrotal lesions [283/638], and 16.4% overall [283/1731]). It was mild in 269 men (95.0%, 269/283), and unilateral in 163 cases (57.6%, 163/283), of which 94 (57.7%, 94/163) were on the right side. Hydrocele was identified more frequently in participants older than 60 years (RR = 1.74, P < 0.05), with diabetes (RR = 2.01, P < 0.05), or with a history of nonspecific urethritis (RR = 1.35, P < 0.05) (Table 2).

Spermatoceles were identified in 174 participants (27.3% of the intrascrotal lesions [174/638], and 10.1% overall [174/1731]). They occurred more frequently in participants older than 60 years (RR = 1.70, P < 0.05), with diabetes (RR = 1.71, P < 0.05), or with no history of vasectomy (RR = 0.77, P < 0.05) (Table 2).

Unilateral testicular hypotrophy/atrophy was present in 167 participants (26.2% of the intrascrotal anomalies [167/638], and 9.7% overall [167/1731]), of which 92 cases (55.1%, 92/167) were on the left side. Bilateral testicular hypotrophy/atrophy was identified in 93 participants (14.6% [93/638] or 5.4% [93/1731]), with complete bilateral atrophy in 13 of them (14.0%, 13/93) (2.0% [13/638] or 0.8% [13/1731]). Hypotrophy/atrophy was identified more frequently in participants older than 60 years. The risk-adjusted prevalence for hypotrophy/atrophy is summarized in Table 2.

Absence of a palpable testis either due to prior orchiectomy or due to cryptorchidism was observed in 20 participants (3.1% of the intrascrotal anomalies [20/638], and 1.2% overall [20/1731]). Prior orchiectomy was reported by 13 men (2.0% [13/638] or 0.8% [13/1731]), of which seven cases (53.8%, 7/13) were on the right side. Cryptorchidism occurred in seven men (1.1% [7/638] or 0.4% [7/1731]), more commonly on the left side (71.4%, 5/7).

Other intrascrotal lesions/anomalies encountered included epididymitis/orchitis in five participants (0.8% [5/638] or 0.3% [5/1731]), with higher prevalence among diabetics than among non-diabetics (RR = 12.90, P < 0.05) (Table 2); postoperative adhesion/hematoma in two cases (0.3% [2/638] or 0.1% [2/1731]); benign paratesticular masses in two cases (0.3% [2/638] or 0.1% [2/1731]); and scrotal calculi in one case (0.2% [1/638] or 0.06% [1/1731]). No testicular cancer was identified in this cohort.

## DISCUSSION

**Scrotal mycosis**

In the present study, scrotal mycosis was identified in 12.1% of all the participants: Tinea cruris in 96.7%, and scrotal tinea in 3.8%.

Tinea of the crural region is an exceedingly common pruritic superficial fungal infection of the groin and adjacent skin including the scrotum.^3^ *Tinea cruris* is more frequent in men than in women, with a 4:1 ratio, and it accounts for 13.9% of all superficial mycotic infections.^4^ It can occur at any age, but is much more common after adolescence due to the hormone-dependent growth of testes and scrotum, enlargement of sweat glands and increasing body weight. High environmental temperature, sweating, prolonged wearing of wet bathing suits and obesity also play a very important role in its causation and repeated relapses.^3^ In our study, the prevalence of *Tinea cruris* was 11.7%, with a 55% higher risk among men with diabetes, and a 32% lower risk among those of nonwhite skin color.

Superficial dermatophytic infection of the scrotum is rare even in the presence of severe infection of the groin and thigh, probably due to the higher pH and the presence of capric acid, one of the fatty acids of the epidermal barrier.^3^ ^,^ ^5^ In our study, the adjusted risk of scrotal tinea was 187% higher among diabetics, and almost six times higher among participants with arterial hypertension, but these results were not significant, most likely due to the low prevalence of this condition.

## Subcutaneous scrotal nodules

Most cases of multiple nodules on the scrotum are due to calcified or noncalcified epidermal cysts, steatocystomas (sebaceous cysts) or other benign tumors.^6^ The term scrotal calcinosis can be used in the presence of calcification, but it has been used inappropriately by many authors even in the absence of calcification.^6^ ^,^ ^7^

The prevalence of subcutaneous nodules in our cohort was 0.7%, with a 334% higher risk among men with elementary school or lower schooling level.

## Hydrocele

The most common cause of scrotal swelling is an accumulation of fluid between the parietal and visceral layers of the tunica vaginalis of the scrotum, surrounding one or both of the testes.^2^ Congenital hydroceles result from a patent processus vaginalis that allows entry of peritoneal fluid into the scrotum. In adults, hydroceles are frequently secondary to trauma, infections (*Bancroftian filariasis*, tuberculosis and sexually transmitted diseases [STDs]), regional or systemic diseases, radiotherapy, inguinal or scrotal surgery or neoplasms, but they are most commonly idiopathic in origin.^2^ ^,^ ^8^

The prevalence of hydroceles differs according to the age group, etiology and evaluation method, with greater frequency of results within the first two years of life, in areas that are endemic for *Bancroftian filariasis* and through routine ultrasound examination. In individuals living in endemic areas of filariasis in the northeastern region of Brazil, hydrocele has been detected through physical examination in 2.2-5.4% of children and adults,^9^ ^-^ ^11^ and 40-50% of men undergoing ultrasound.^12^ In non-endemic areas, the clinical prevalence of hydrocele ranges from 0.6 to 2.9%,^10^ and the ultrasound prevalence is around 25-35%.^13^ ^,^ ^14^

In our study, although a small amount of hydrocele was detected through meticulous scrotal palpation in 15.5% of the participants > 40 years of age in a non-endemic region, moderate to severe hydrocele was identified through inspection or palpation in only 0.8%.

The risk of hydrocele was 35% higher in subjects with histories of nonspecific urethritis. STDs may be associated with secondary involvement of the epididymis, which may potentially cause defective drainage or obstruction of the venous/lymphatic vessels of the tunica vaginalis.^7^ ^,^ ^15^ In these cases, the resultant hydrocele may persist even after the triggering factor has been treated.^15^

The risk-adjusted prevalence of hydroceles among diabetics was twice as high among the participants of our study. In the presence of diabetes *mellitus*, development of hydroceles may result from microvascular disease and occlusion, or from increased susceptibility to epididymitis.^16^

## SPERMATOCELES

Spermatoceles are the most common cystic condition encountered within the scrotum. They are usually situated in the head of the epididymis, frequently asymptomatic, occasionally bilateral and multiple, and often smaller than one centimeter.^17^

It has been estimated that about 30% of asymptomatic men undergoing scrotal ultrasound have one or more of these cysts.^8^ ^,^ ^18^ Many urologists are comfortable with making a diagnosis based on history and physical examination alone, while others often use ultrasound to confirm it.^8^ However, imaging studies do not provide additional information in men with spermatocele in whom the testes are palpably normal.^19^

Cystic dilatations of the tubules of the efferent ductules of the epididymis probably result from obstruction, and are more frequently encountered in cases of cystic fibrosis, von Hippel-Lindau disease, maternal exposure to diethylstilbestrol and polycystic kidney disease.^7^ ^,^ ^17^

In our cohort, spermatoceles were clinically detected in around 10% of the subjects, with a 70% higher prevalence in men > 60 years of age, and a similarly increased risk among diabetics. Although diabetes *mellitus* is not usually reported as a risk for spermatoceles, diabetes is an acknowledged risk factor for renal, pancreatic and ovarian cysts, and it is also more frequently correlated with cystic fibrosis and polycystic kidney disease, which are often associated with spermatoceles.^7^ ^,^ ^17^

We also identified an inverse relationship between spermatoceles and vasectomy. However, since most participants with histories of vasectomy were < 60 years of age, and spermatoceles were more prevalent in men > 60 years, it may be possible that the inverse relationship may have been biased by the lower age of the participants with histories of vasectomy.

## Testicular hypotrophy/atrophy

Hypotrophy (hypoplasia) and atrophy of the testes refers to partial or complete shrinking of testicular volume. The prevalence of testicular hypotrophy/atrophy is dependent on the underlying cause of the condition.^20^ ^-^ ^22^ In our cohort of men attending a prostate cancer screening program, the prevalences of unilateral and bilateral testicular hypotrophy/atrophy were, respectively, 9.7% and 5.4%.

A variety of reports have suggested that testicular volume declines with advancing age.^23^ In the present study, the prevalence of both unilateral and bilateral hypotrophy/atrophy was roughly 60% higher among men > 60 years of age. Other independent risk factors for unilateral testicular hypotrophy/atrophy include testicular torsion, cryptorchidism, nonspecific orchitis, mumps orchitis, genital trauma, varicocele, surgical injury, or a combination of these factors. On the other hand, bilateral testicular hypotrophy/atrophy may be caused by malnutrition, alcohol and drug abuse and numerous chronic illnesses, although most cases are idiopathic, and several causes of unilateral atrophy may result in contralateral testicular injury.^20^ ^,^ ^21^ ^,^ ^23^ ^-^ ^25^

The subjects with histories of vasectomy showed a 59% lower risk of bilateral hypotrophy/atrophy, compared with those without a history of vasectomy. Although this result was statistically significant and it may imply that vasectomy protects against potential risk factors for bilateral testicular hypotrophy/atrophy, it is possible that this was confounded by uncontrolled factors. It is plausible, for example, that men who developed bilateral testicular failure already had a history of infertility or subfertility when they were younger and, therefore, were not considered eligible for vasectomy.

## Epididymitis/orchitis

Epididymitis, the most common cause of acute scrotal pain in all age groups, is usually caused by bacterial reflux from the bladder or the prostate gland through the vas deferens to the epididymis, and it often spreads to the testes (epididymo-orchitis).^2^ Isolated orchitis is rare and it is generally associated with viral infections.^26^

Epididymitis/orchitis occurs in approximately 0.7% of men aged 18 to 50 years,^26^ and it occurred in 0.3% of the men > 40 years of age in our study. A bimodal distribution has been observed, with peak incidences occurring among men aged 16 to 30 years and among those aged 51 to 70 years.^26^ In sexually active men younger than 35 years of age, chlamydia and gonorrhea are the most common causes. In men older than 35 or those who practice insertive anal intercourse, enteric Gram-negative bacilli are the most common causative pathogens.^2^ ^,^ ^26^

The risk factors include sexual activity, bladder outlet obstruction, recent urinary tract surgery or instrumentation, anatomical anomalies, strenuous physical activity, bicycle or motorcycle riding, and prolonged periods of sitting.^2^ ^,^ ^26^ We also identified a higher risk of epididymitis/orchitis among participants with diabetes *mellitus*, who presented a nearly 13-fold higher risk.

## Cryptorchidism

Testicular ectopy comprises failure of the testes to descend completely, unilaterally or bilaterally into the scrotum, and it is the most prevalent congenital anomaly at birth. The prevalence of cryptorchidism is age-dependent, with a 9.2-30% prevalence among premature infants, 3.4-5.8% among full-term infants, 0.8-1.82% at one year of age, and 0.8-1% at puberty and into adulthood.27 Nonpalpable testes accounts for only 20% to 30% of the cases.2 In the present study, nonpalpable testes due to cryptorchidism were registered in 0.5% of the men > 40 years of age, with an almost threefold higher prevalence (but without statistical significance) among participants with low schooling level.

## Limitations of the study

This epidemiological study only involved men > 40 years of age within an established private healthcare system, but it covers a specific age range of adults that provides important information about the prevalence and risk factors of several scrotal diseases in this group.

The participants were examined in the supine position. By doing so, we failed to detect prevalent intrascrotal lesions, including varicoceles and inguinal hernias, which are better evaluated in the standing-up position. Furthermore, experimental and clinical observations have shown substantial imprecision in clinical evaluations and measurements on the testes.^23^

## Implications for clinical practice and future research

The results from our study are important as an epidemiological resource for patients and healthcare providers, and as a means of comparison with other populations. Our results have established associations between several epidemiological risk factors that were hitherto poorly evaluated in the literature and a variety of scrotal lesions/anomalies routinely seen in consultation office practice. Future studies should validate the consistency of the associations identified in our study.

## CONCLUSIONS

We estimated the prevalence of scrotal diseases commonly seen in consultation office practice, in an adult population of Brazilian men. The prevalence of cutaneous lesions was 13.3%, and mycosis was present in most of these (91.7%). We found significant correlations linking Tinea cruris with diabetes and white skin color; scrotal tinea with arterial hypertension; subcutaneous nodules with low schooling level and no history of nonspecific urethritis; hydrocele with age older than 60 years, diabetes and histories of nonspecific urethritis; spermatoceles with age older than 60 years, diabetes and no history of vasectomy; testicular hypotrophy/atrophy with age older than 60 years; and epididymitis/ orchitis with diabetes.
